# Spatial analysis of the incidence of Dengue, Zika and Chikungunya and socioeconomic determinants in the city of Rio de Janeiro, Brazil

**DOI:** 10.1017/S0950268821001801

**Published:** 2021-08-02

**Authors:** Eny Regina da Silva Queiroz, Roberto de Andrade Medronho

**Affiliations:** 1 Institute for Studies in Collective Health, Federal University of Rio de Janeiro (Universidade Federal do Rio de Janeiro – UFRJ), Rio de Janeiro, RJ – Brazil; 2 Faculty of Medicine, Institute for Studies in Collective Health, Federal University of Rio de Janeiro (Universidade Federal do Rio de Janeiro – UFRJ), Rio de Janeiro, RJ – Brazil

**Keywords:** Arboviruses, chikungunya, dengue, spatial analysis, Zika

## Abstract

In 2015–2016, simultaneous circulation of dengue, Zika and chikungunya in the municipality of Rio de Janeiro (Brazil) was reported. We conducted an ecological study to analyse the spatial distribution of dengue, Zika and chikungunya cases and to investigate socioeconomic factors associated with individual and combined disease incidence in 2015–2016. We then constructed thematic maps and analysed the bivariate global Moran indices. Classical and spatial models were used. A distinct spatial distribution pattern for dengue, Zika and chikungunya was identified in the municipality of Rio de Janeiro. The bivariate global Moran indices (*P* < 0.05) revealed negative spatial correlations between rates of dengue, Zika, chikungunya and combined arboviruses incidence and social development index and mean income. The regression models (*P* < 0.05) identified a negative relationship between mean income and each of these rates and between sewage and Zika incidence rates, as well as a positive relationship between urban areas and chikungunya incidence rates. In our study, spatial analysis techniques helped to identify high-risk and social determinants at the local level for the three arboviruses. Our findings may aid in backing effective interventions for the prevention and control of epidemics of these diseases.

## Introduction

Arboviruses represent a significant global public health challenge, having emerged in and spread to many countries in recent decades. In 2020, 2 350 286 cases of dengue, Zika and chikungunya were reported in the Americas. Among these cases, 64.5% were reported in Brazil [1].

Two emerging arboviruses transmitted by the same vector, *Aedes aegypti*, are Zika virus fever and chikungunya fever. Until the 2000s, these diseases were considered rare and were circumscribed to a few locations worldwide. They later spread to various countries, causing important epidemics. Chikungunya can cause incapacitating and prolonged symptoms, especially in the joints, while Zika is associated with congenital malformation syndrome, which includes microcephaly [2].

Since the re-emergence of dengue in the 1980s in Brazil [3], the city of Rio de Janeiro experienced alternating endemic and epidemic periods. Autochthonous cases of chikungunya and Zika, and simultaneous circulation of the three arboviruses in the city were reported for the first time in 2015 [4].

The proliferation of the *A. aegypti* vector is affected by ecological and socioenvironmental factors. High temperatures, which are typical of the summer, and extend into part of autumn in Rio de Janeiro, can accelerate mosquito egg development and increase the adult population. The human population concentration in urbanised areas with precarious sanitation conditions favours the emergence of potential mosquito breeding sites [4, 5].

Spatial analysis techniques have been used to investigate spatial and spatiotemporal patterns for dengue, Zika and chikungunya and associated risk factors in different locations and scales [5–8]. However, there are still gaps in the knowledge of these factors in the context of the simultaneous occurrence of the three arboviruses on the local scale.

The present study aimed to analyse the spatial distribution of dengue, Zika and chikungunya cases in 2015−2016 and to identify associated socioeconomic factors for each of these diseases. We also aimed to analyse the three arboviruses jointly to investigate the spatial dynamics of diseases transmitted by a single vector, *A. aegypti*, in the territory.

## Methods

This cross-sectional ecological, epidemiological study analysed data for areas demarcated according to neighbourhoods.

### Location and study period

**Fig. 1.**
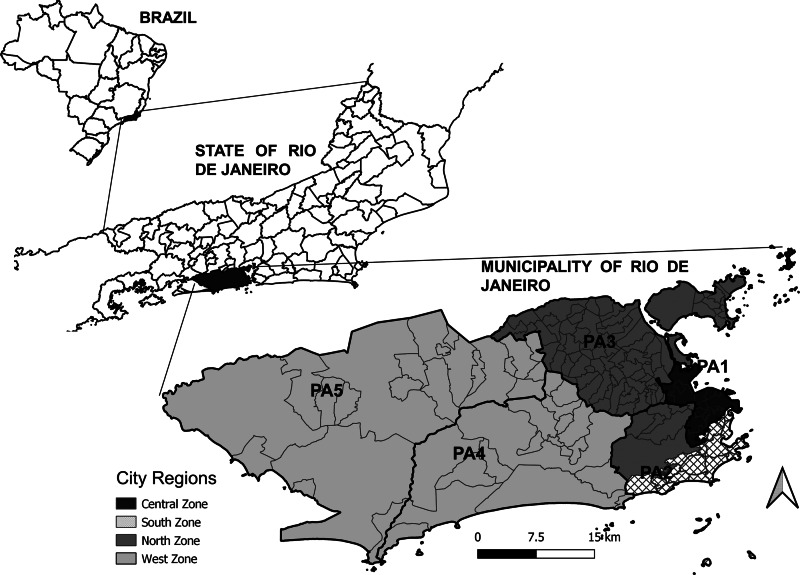
Maps of Brazil with divisions by state, the State of Rio de Janeiro with divisions by municipality, and the Municipality of Rio de Janeiro with divisions by region (legend) and planning areas (PA, codes).

The study area consisted of the city of Rio de Janeiro, the capital of the state of Rio de Janeiro, located in the southeast region of Brazil, which is situated at 23°04′10″ S and 43°47′40″ W. With 6 320 446 inhabitants in 2010, Rio de Janeiro is the second-largest city in Brazil and is home to 39.5% of the population in the state with the same name (Fig. 1).

Rio de Janeiro is marked by social and economic heterogeneities, with economically more privileged areas surrounded by precarious housing conditions and deficient sanitation infrastructure.

The city is divided into five planning areas (PAs), 33 administrative regions, and 160 neighbourhoods (Fig. 1). Historically, the city has four main regions: the North Zone, South Zone, Central Zone, and West Zone (Fig. 1). The Central Zone (PA 1), which includes the historical and downtown parts of the city, underwent major urban development over the years and includes a large commercial and business area. The North Zone (PA 2.2 and PA 3) is the most heavily populated part of the city, with slightly more than half of the city's neighbourhoods and 42% of the population. The South Zone (PA 2.1) is located close to the Central Zone, between the Atlantic Ocean and the Tijuca Massif. The South Zone's neighbourhoods have the highest Social Development Indices (SDI). The West Zone (PA 4 and PA 5) is the most recently occupied area of the city with the lowest population density, occupying the largest portion of the city's territory. Neighbourhoods with lower SDI are predominant in the West and North Zones [9].

The study period was restricted from the epidemiological week (EW) 44/2015 to EW 34/2016, as we aimed to cover phases in the growth and decline in the number of reported cases of the three diseases based on the date of the symptoms' onset.

### Data source

Socioeconomic and demograph data were obtained from the 2010 Nationl Census performed by the Brazilian Institute of Geography and Statistics, which is available by neighbourhoods through the official open data platform of the government of the municipality of Rio de Janeiro (DATA.RIO, (http://www.data.rio/). The digital map grids population was also extracted from DATA.RIO.

Data related to income and basic sanitation conditions produced by the Census have already been used as indicators of economic and social aspects of populations in ecological studies on disease risk factors [8, 10].

We selected a set of variables that reflected infrastructure, basic sanitation, socioeconomic status, population density, and urbanisation according to the neighbourhood: percentage of households connected to the public water supply (WATER), percentage of households with adequate sewage disposal (SEWAGE), percentage of households with public garbage collection (GARBAGE COLLECTION), social development index (SDI), population density (POPULATION DENSITY), percentage of the urban area (URBAN AREA) and mean income of the head-of-household (MEAN INCOME). The SDI is a composite indicator inspired by the human development index (HDI), which add information regarding basic sanitation, housing quality, schooling and income to more appropriately reflect the neighbourhood's social and urban characteristics [9].

The study utilised arboviruses data contained within the Brazilian National Notifiable Diseases Information System (Sistema de Informação de Agravos de Notificação (SINAN)). SINAN data have been used in many epidemiological studies [10–13]. Cases included in the national list of compulsory notifications are investigated and recorded in this system at the municipal level. SINAN data provided by the municipal epidemiological surveillance service, aggregated by neighbourhood and by epidemiological weeks were extracted from the following website: http://www.rio.rj.gov.br/web/sms/vigilancia-epidemiologica-da. The study covered all reported cases of dengue, Zika and chikungunya excluding those occurring in neighbourhoods that could not be identified.

### Statistical methods

The crude mean incidence rates were calculated as cases per hundred thousand inhabitants. The local empirical Bayesian method was used, including information from neighbouring areas to estimate each spatial unit's smoothed rate [14]. This procedure aims to reduce the effect of possible underreporting in spatial units. In addition to the incidence rates for Zika, dengue and chikungunya, the combined arboviruses incidence rates were calculated by inserting the sum of cases for the numerator's three diseases. To approximate a normal distribuition, log transformations of the smoothed incidence rates and covariables were used for regression models.

The exploratory stage involved building thematic maps of incidence rates and calculating the univariate global Moran indices for the crude incidence rates and bivariate global indices between the smoothed rates and covariables. Maps were made using QGIS 3.10 [15] and the Moran indices were calculated using GeoDa 1.12.1.59 [16].

The criterion stipulated by the National Dengue Control Plan was used to characterise the spatial distribution of arboviruses. This criterion, classifies the incidence rates (i.e. cases per hundred thousand inhabitants) as low (0−100 cases), average (101−299), and high (300 cases or more) [17]. For the construction of thematic maps, a fourth category reflecting more than 700 cases per hundred thousand inhabitants was created.

Spatial autocorrelation indices indicate the degree of association between a variable's measure and its localisation. They express the degree of similarity between each unit and its neighbours. The global Moran I coefficient varies from −1 to +1. Positive values indicate that neighbouring areas are similar to the mean of the target variable's values in the entire study area. Negative values indicate negative autocorrelation, and null values indicate the absence of spatial dependence. Bivariate Moran I measures the spatial dependence of the covariance between two attributes [18].

Classical and spatial models were then developed. Variables exhibiting statistically significant coefficients in the bivariate analyses, for each outcome were selected for the initial models. A Spearman correlation matrix was constructed for the independent variables. When two variables exhibited a correlation equal to or greater than 0.5, with statistical significance, only one was included in the model.

Linear regression models were built for target outcomes with the selected covariables. Next, a test diagnosis of multicollinearity variance inflation factor (VIF) was performed. The Moran index was calculated to assess the autocorrelation of residuals.

The spatial error or conditional auto-regressive (CAR) model was also used for the incidents rates with the covariables. This model is a global spatial model that captures the spatial dependence structure in a parameter added to the traditional regression model. In this method, spatial dependence is incorporated into the error's structure, and the spatial effects are considered something to be removed:

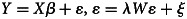

where *λ* is the autoregressive coefficient, *Wε* is the component of the error with spatial effects, and *ξ* is the component of the error with constant and non-correlated variance, under the null hypothesis of absence of spatial dependence (i.e. *λ* = 0) [19].

Another class of CAR model was also used, following the Bayesian hierarchical approach. The models were estimated under the assumption that the number of cases of dengue, Zika, chikungunya and the three combined arboviruses follows the Poisson distribution and the population was considered as an offset [20].

The variables and interaction terms were gradually maintained or discarded according significance of the coefficients (*P* < 0.05). For the final model's definition, all the linear regresson models and the two spatials models were compared, considering the Akaike information criterion (AIC) and the Moran Index of the residuals.

Regression analysis was performed using RStudio 1.1.463 [21] with the following packages of the R software, version 4.0.5 [22]: rgdal, sp, spdep, faraway, spatialreg and CARBayess.

## Results

During the study period, there were 26 816 cases of dengue, 38 413 cases of Zika, and 13 624 cases of chikungunya. Among the reported cases, 2060 (7.7%) cases of dengue and 37 (0.01%) cases of Zika were excluded because the neighbourhoods were not identified.

**Fig. 2.**
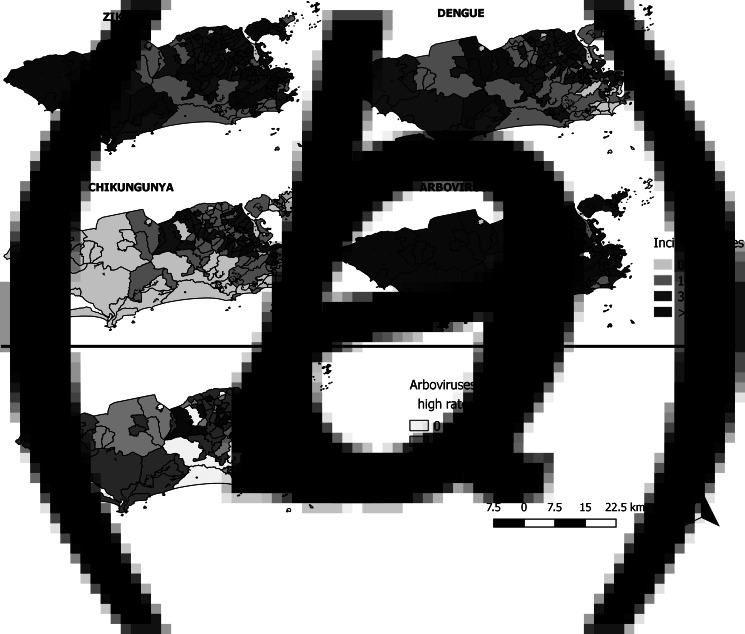
**A** – Maps of incidence rates for dengue, Zika and chikungunya. **B** – Number of arboviruses with rates greater than 300 cases per 100 000 inhabitants in each neighbourhood. City of Rio de Janeiro, RJ, Brazil. Epidemiological week 44/2015 to 34/2016.

High Zika incidence rates (more than 300 cases per 100 000 inhabitants) were observed in all city areas. The highest dengue incidence rates were in neighbourhoods in the West Zone and North Zone, while the highest chikungunya incidence rates were in the North Zone and some neighbourhoods in the Central Zone (Fig. 2A). Despite the occurrence of a triple epidemic at the municipal level, only 25% of the neighbourhoods simultaneously exhibit high dengue, Zika and chikungunya incidence rates (Fig. 2B).

The global Moran indices indicated a statistically significant spatial autocorrelation (*P* < 0.05) for incidence rates of Zika, chikungunya and the three arboviruses combined, meaning that adjacent neighbourhoods tended to have similar incidence rates (Table 1).

**Table 1.** Global Moran indices (*P* values in brackets) for incidence rates of Zika, dengue, chikungunya and the three arboviruses combined

	Zika	Dengue	Chikungunya	The three arboviruses
**Univariate Moran** (with crude rates)	0. 12 (0.02)	0.06 (0.07)	0.34 (0.00)	0.13 (0.01)
**Bivariate Moran** (with smoothed rates)	Zika	Dengue	Chikungunya	The three Arboviruses
Mean income	−0.12 (0.00)	−0.14 (0.00))	−0.20 (0.00)	−0.18 (0.00)
Social development index − SDI	−0.22 (0.00)	−0.13 (0.00)	−0.09 (0.01)	−0.20 (0.00)
Sewage	−0.29 (0.00)	0.02 (0.27)	0.19 (0.00)	−0.11 (0.01)
Garbage collection	−0.02 (0.22)	−0.02 (0.24)	−0.04 (0.16)	−0.04 (0.16)
Water	−0.17 (0.00)	0.01 (0.45)	0.11 0.01	−0.05 (0.08)
Urban area	−0.04 (0.07)	0.03 (0.24)	0.26 (0.00)	−0.06 (0.44)
Population density	−0.13 (0.00)	−0.01 (0.45)	0.13 (0.00)	−0.03 (0.17)

City of Rio de Janeiro, RJ, Brazil. Epidemiological week 44/2015 to 34/2016.

The bivariate global Moran indices indicated that the mean Zika incidence rate exhibited negative spatial correlations with SDI, MEAN INCOME, SEWAGE, WATER and POPULATION DENSITY. The dengue incidence rates exhibited a negative spatial correlations with SDI, MEAN INCOME and POPULATION DENSITY. The chikungunya incidence rates exhibited negative spatial correlations with SDI, MEAN INCOME, as well as positive spatial correlations with SEWAGE, WATER, URBAN AREA and POPULATION DENSITY. The combined arboviruses incidence rates exhibited negative spatial dependence with SDI, MEAN INCOME and SEWAGE. Notably, the dengue, Zika and chikungunya incidence rates and the combined incidence rates of the three arboviruses exhibited a negative spatial association with MEAN INCOME and SDI (Table 1).

The Spearman correlation matrix of the independent variables identified a significant correlation between SDI and MEAN INCOME, between SDI and SEWAGE, between SEWAGE and WATER, and between POPULATION DENSITY and URBAN AREA (Table 2). The coefficients of the bivariate models were listed in Table 3.

**Table 2.** Spearman' correlation matrix (*P* values in brackets)

	Social development index - SDI	Sewage	Mean income	Garbage collection	Water	Urban area	Population density
Social development index - SDI	1.0						
Sewage	0.64 (0.0)	1.0					
Mean income	0.87 (0.0)	0.39 (0.0)	1.0				
Garbage collection	0.19 (0.01)	0.06 (0.40)	0.13 (0.05)	1.0			
Water	0.40 (0.0)	0,62 (0,0)	0.19 (0.01)	0.11 (0.15)	1.0		
Urban area	0.11 (0.17)	0.39 (0.0)	−0.13 (0.1)	0.13 (0.1)	0.41 (0.01)	1.0	
Population density	0.17 (0.02)	0.43 (0.0)	−0.05 (0.5)	−0.12 (0.13)	0.40 (0.0)	0.65 (0.0)	1.0

**Table 3.** Regression coefficients and confidence intervals of the bivariate models

	Zika			Dengue			Chikungunya			Arboviruses		
	Linear regression	CAR	Poisson	Linear regression	CAR	Poisson	Linear regression	CAR	Poisson	Linear regression	CAR	Poisson
Mean income	−0.22 (−0.42, −0.02)	−0.24 (−0.46, −0.03)	−0.05 (−0.1, −0.02)	−0.49 (−0.7, −0.3)	−0.43 (−0.66, −0.19)	−0.10 (−0.14, −0.06)	−0.56 (−0.8, −0.3)	−0.48 (−0.72, −0.24)	−0.14 (−5.1, −5.5)	−0.4 (−0.6, −0.2)	−0.37 (−0.58, −0.17)	−0.08 (−0.13, −0.04)
Social development index - SDI	−1.58 (−2.7, −0.5)	−1.39 (−2.5, −0.27)	−3.18 (−5.18, −1.41)	−2.59 (−3.8, −1.4))	−2.10 (−3.37, −0.81)	−5.63 (−8.3, −2.6)	−1.48 (−2.8, −0.1)	−1.74 (−3.04, −0.43)	−6;22 (−9.28, −3.5)	−1.96 (−3.0, −0.9)	−0.20 (−3.09, −0.99)	−3.84 (−5.42, −1.47)
Sewage	−0.87 (−1.5, −0.2)	−0.76 (−1.4, 0.07)	−0.004 (−0.01, 0.01)	−0.66 (−1.4, 0.1)	0.36 (−1.21, 0.48)	0.0 (−0.02, 0.03)	1.05 (0.3, 1.8)	0.16 (−0.71, 1.03)	0.01 (−0.05, 0.03)	0.6 (1.2, 0.1)	−0.53 (−1.22, 0.15)	−0.01 (−0.02, 0.01)
Garbage collection	−0.33 (−0.9, 0.2)	−0.45 (−1.0, 0.1)	−0.01 (−0.02, 0.0)	−0.44 (−1.1, 0.2)	−0.68 (−1.30, −0.06)	−0.01 (−0.02, −0.002)	−0.15 (−0.8, 0.5)	−0.32 (−0.93, 0.28)	0.0 (−0.01, 0.01)	−0.38 (−0.9, 0.2)	−0.50 (−1.07, 0.07)	−0.01 (−0.02, 0.0)
Water	−0.37 (−0.9; 0.2)	−0.10 (−0.7, −0.5)	−0.01 (−0.03, 0.01)	−0.28 (−0.98, 0.42)	−0.10 (−0.76, 0.56)	0.02 (0.02, 0.04)	0.54 (−0.2, 1.3)	0.02 (−0.61, 0.65)	0.0 (−0.03, 0.04)	−0.16 (−0.2, 1.3)	−0.03 (−0.53, 0.35)	002 (−0.01, 0.05)
Urban area	−0.03 (−0.2, 0.2)	0.07 (−0.14, 0.30)	0.004 (−0.01, 0.01)	0.03 (−0.2, 0.3)	0.14 (−0.10, 0.40)	0.01 (0.007, 0.012)	0.50 (0.27, 0.72)	0.31 (0.07.;0.56)	0.01 (0.001, 0.02)	0.1 (0.1, 0.3)	0.12 (−0.08, 0.34)	0.0 (0.002;0.005)
Population density	0.1 (−0.2, 0.0)	−0.05 (−0.17, 0.06)	0.001 (−0.0001, 0.002)	−0.03 (−0.2, 0.1)	0.01 (−0.13, 0.14)	0.0 (0.0, 0.00)	0.20 (0.1, 0.3)	0.07 (−0.06, 0.21)	0.0 (−0.001, 0.002)	−0.3 (−0.1, 0.1)	−0.01 (−0.12. 0.11)	0.0 (−0.002;0.001)

Poisson: spatial Bayesian Poisson; CAR: conditional auto-regression.

Epidemiological week 44/2015–34/2016 (Rio de Janeiro, RJ, Brazil).

The estimated CAR models for the dengue and chikungunya incidence rates vielded a lower AIC than the linear regression models. AIC was similar for the CAR and linear regression models for Zika incidence rates, but the Moran index indicated autocorrelation of the residuals of the linear model. Zika incidence rates were negatively associated with MEAN INCOME and SEWAGE. Dengue incidences were negatively associated with MEAN INCOME. Chikungunya incidence rates were positively associated with URBAN AREA, and negatively associated with MEAN INCOME. The adjusted classic model for the incidence rates of the combined arboviruses revealed a negative association between incidence rates of the three arboviruses combined and MEAN INCOME, and the AIC was lower for the linear regression model than for the CAR model. For all outcomes, the AIC of the Poisson models was higher than that of the other models. The residuals Moran Index of the final models did not indicate spatial dependence (Tables 4 and 5).

**Table 4.** Final models, according to the AIC criterion and absence of autocorrelation of residues, for Dengue, Zika and Chikungunya and combined arbovirus incidence rates and covariates

	Variables	Coefficients	CI (95%)
**Zika** **CAR** AIC = 371.2 *I* = 0.0 *P* = 0.182	Mean income	−0.22	(−0.43, −0.01)
	Sewage	−0.70	(−1.38, −0.03)
**Dengue** CAR AIC = 407.2 *I* = −0.01 *P* = 0.42	Mean income	−0.43	(−0.66, −0.19)
**Chikungunya** CAR AIC = 394.1 *I* = −0.001, *P* = 042	Mean income	−0.49	(−0.72, −0.26)
	Urban area	−0.33	(0.10, 0.56)
**Arboviruses** LM AIC = 375 *I* = 0.0 *P* = 0.28	Mean income	−0.37	(−0.58, −0.18)

Epidemiological week 44/2015–34/2016 (Rio de Janeiro, RJ, Brazil).

**Table 5.** Final models between incidence rates (linear regression and CAR) or number of cases (spatial Bayesian Poisson) of Zika, Dengue and Chikungunya and covariates obtained using three different approaches

	Linear regression		CAR		Spatial Bayesian Poisson	
**Zika**	Mean income^a^	−0.20 (−0.40, −0.003)	Mean income	−0.22 (−0.43, −0.01)	SDI	−3.18 (−5.18, −1.41)
	Sewage^a^	−0.81 (−1.43, −1.18)	Sewage	−0.70 (−1.38, −0.03)		
	AIC = 371.6		AIC = 371.2		AIC = 1376.84	
	*I*−0.04, *P* = 0.02		*I* = 0.0, *P* = 0.182		*I* = −0.0, *P* = 0.42	
**Dengue**	Mean income	−0.49 (−0.71, −0.27)	Mean income	−0.43 (−0.66, −0.19)	Mean income	−0.10 (−0.14, −0.06)
	AIC = 410.87		AIC = 407.2			AIC = 1282.7
			*I* = −0.01, *P* = 0.42			*I* = 0.02, *P* = 0.08
**Chikungunya**	Mean income^a^	−0.52 (−0.74, −0.29)	Mean income	−0.49 (−0.72, −0.26)	SDI	−4.85 (−7.39, −1.92)
	Urban area^a^	0.46 (0.24, 0.67)	Urban area	−0.33 (0.10, 0.56)	Urban area	0.016 (0.01, 0.02)
	AIC = 408.98		AIC = 394.1		AIC = 1184.8	
	*I* = 0.16 *P* = 0.00		*I* = −0.001, *P* = 042		*I* = 0.01, *P* = 0.1	
**Arboviruses**	Mean income	−0.37 (−0.58, −0.18)	Mean income	0.37 (−0.57, −0.17)	SDI	−3.84 (−5.42, −1.46)
	AIC = 375		AIC = 377		AIC = 1509.9	
	*I* = 0.0, *P* = 0.28		*I* = 00, *P* = 0.39		*I* = −0.2, *P* = 0.73	

Epidemiological week 44/2015–34/2016 (Rio de Janeiro, RJ, Brazil).

a VIF < 1.1.

## Discussion

In the present study, we conducted a spatial analysis of the incidence of dengue, Zika and chikungunya and their socioeconomic determinants in the city of Rio de Janeiro, Brazil. Our analysis identified a distinct spatial distribution pattern for dengue, Zika and chikungunya; the first time a triple epidemic of these arboviruses was recorded in Rio de Janeiro. The analysis also revealed a spatial relationship between low economic status and higher incidence rates of arboviruses.

Zika incidence rates presented greater spatial dispersion and Zika was present in all regions of the city. In most neighbourhoods, there were no simultaneous records of high dengue, Zika and chikungunya incidence rates, which may indicate that the circulation of one arbovirus partially inhibited the circulation of the other two arboviruses. This finding is an accordance with those of previous R studies, such as another conducted in the city of Rio de Janeiro, in which clusters of the three arboviruses did not coincide in time and space due to competition between the viruses or vector control activities conducted in previous outbreaks [13]. A study in the Dominican Republican also reported asynchronous spatial and temporal characteristics of the three arboviruses. The authors suggested that previous immunity to any of the arboviruses or problems in confirming the diagnosis may have contributed to this finding [23]. Another study conducted in Salvador (Bahia, Brazil), reported a reduction in the detection of positive cases in laboratory tests for dengue in patients with acute febrile syndrome during and immediately after a Zika epidemic. The authors hypothesised that Zika infection conferred immunity to dengue [24].

In contrast, a previous ecological study conducted in Brazil evaluated the effect of previous immunity against dengue on the occurrence of microcephaly due to Zika. The authors estimated that, when the dengue epidemic occurred up to 6 years before the Zika epidemic, this conferred a protective effect against microcephaly. However, the occurrence of a Zika epidemic 7–12 years after a dengue epidemic increased the risk of microcephaly [25].

Fuller *et al*. [6] reported spatial juxtaposition of Zika and chikungunya in cities within the state of Rio de Janeiro. According to the authors, the increase in the incidence rate of chikungunya began after the decline in Zika incidence, indicating possible competition between the arboviruses in the vector.

In the exploratory phase of the current study, spatial dependence analysis indentified a spatial relationship between low SDI, low income and higher Zika, dengue chikungunya incidence rates. Spatial modelling corroborated the importance of socioeconomic factors and sanitation conditions in the spatial distribution of each of these rates. Other studies have demonstrated an association between low income indicators and a higher risk of dengue [8, 12, 26]. These findings emphasise the need for specific interventions to contain epidemics and promote health in less economically favoured regions that present with complex realities, such as geographical constraints and public security problems. However, other studies have reported conflicting results regarding the association between indicators of socioeconomic vulnerability and dengue incidence, suggesting that this relationship requires more in-depth investigation [27, 28].

The spatial relationship between inadequate sewage disposal and Zika incidence rates is consistent with previous dengue results. Sewage disposal services can be considered a proxy for sanitation conditions, including water supply and garbage collection. These findings highlight for intervention targeting these factors [7, 10].

The current study also revealed an association between a high percentage of urban area and higher chikungunya incidence rates. Studies suggest that high population density and high proportion of urbanisation are associated with higher dengue incidence rates because they provide favourable conditions for the proliferation of the disease's vector, which is heavily adapted to household environments [7, 29, 30].

This study had some limitations. The use of secondary data aggregated by neighbourhoods with arbitrary limits in this study may not have captured local nuances and heterogeneities. Topological aspects and transport networks, and urban mobility were not considered. The lack of laboratory confirmation in this study, which involved three diseases with similar symptoms, the emphasis of the epidemiological surveillance service on Zika virus infections, declared as Public Health Emergency of International Concern in 2016 may have favoured the occurrence of information bias. However, the analysis of the joint incidence rates for all three arboviruses allowed the spatial investigation of these diseases transmitted by the same vector in the city.

In this ecological study, spatial analysis techniques were used to identify areas with a more intense circulation of symptomatic cases of these three important arboviruses in the first occurrence of a triple epidemic. These techniques thus allow identifying areas at risk and social determinants of the occurrence of these diseases in any place. These findings may help to subside the choice of priority areas for the allocation of health surveillance and assistance resources.

Importantly, increasing the knowledge on each of these three arboviruses' spatial dynamics and their determinants at the local level is essential for backing effective interventions in the prevention and control of their epidemics.

## Data Availability

The data that support the findings of this study are available from http://www.rio.rj.gov.br/web/sms/vigilancia-epidemiologica-da.

## Supplementary material

For supplementary material accompanying this paper visit http://dx.doi.org/10.1017/S0950268821001801. click here to view supplementary material
