# Supplementary material for: Spatial analysis of the incidence of Dengue, Zika and Chikungunya and socioeconomic determinants in the city of Rio de Janeiro, Brazil
Source: Epidemiol Infect. 2021 Aug 2;149:e188. doi: 10.1017/S0950268821001801 (PMC8365848; doi:10.1017/S0950268821001801)
Supplement: Supplementary file 1 [file S0950268821001801sup001.doc]

**S1. STROBE checklist.**

|  | Item No | Recommendation |
| --- | --- | --- |
| **Title and abstract** | 1 | (*a*) Indicate the study’s design with a commonly used term in the title or the abstract – *Page 2 “an ecological study to analyze the spatial distribution of dengue, Zika, and chikungunya cases and to investigate socioeconomic factors associated with individual and combined disease incidence in 2015–2016.”* (Abstract) |
|  |  | (*b*) Provide in the abstract an informative and balanced summary of what was done and what was found – *Page 2* |
| Introduction | | |
| Background/rationale | 2 | Explain the scientific background and rationale for the investigation being reported – *Pages 3* |
| Objectives | 3 | State specific objectives, including any prespecified hypotheses – *Page 4 “The present study aimed to analyze the spatial distribution of dengue, Zika, and chikungunya cases in 2015-2016 and to identify associated socioeconomic factors for each of these diseases. We also aimed to analyze the three arboviruses jointly to investigate the spatial dynamics of diseases transmitted by a single vector, Aedes aegypti, in the territory.”* |
| Methods | | |
| Study design | 4 | Present key elements of study design early in the paper – *Pages 4-8* |
| Setting | 5 | Describe the setting, locations, and relevant dates, including periods of recruitment, exposure, follow-up, and data collection – *Pages 4-6* |
| Participants | 6 | (*a*) Give the eligibility criteria, and the sources and methods of selection of participants – *Pages 6* |
| Variables | 7 | Clearly define all outcomes, exposures, predictors, potential confounders, and effect modifiers. Give diagnostic criteria, if applicable – *Page 5-6;8* |
| Data sources/ measurement | 8* | For each variable of interest, give sources of data and details of methods of assessment (measurement). Describe comparability of assessment methods if there is more than one group – *Page 5-6* |
| Bias | 9 | Describe any efforts to address potential sources of bias – *N/A* |
| Study size | 10 | Explain how the study size was arrived at – *Page 5-6* |
| Quantitative variables | 11 | Explain how quantitative variables were handled in the analyses. If applicable, describe which groupings were chosen and why – *Page 5- 6* |
| Statistical methods | 12 | (*a*) Describe all statistical methods, including those used to control for confounding – *Page 6-8* |
|  |  | (*b*) Describe any methods used to examine subgroups and interactions – *Pages 7-8* |
|  |  | (*c*) Explain how missing data were addressed –*Any relevant missing data were listed in the paper. These were minimal and were not included in any analysis* - Page 6 |
|  |  | (*d*) If applicable, describe analytical methods taking account of sampling strategy – *N/A* |
|  |  | (*e*) Describe any sensitivity analyses – *N/A* |
| Results | | |
| Participants | 13* | (a) Report numbers of individuals at each stage of study—eg numbers potentially eligible, examined for eligibility, confirmed eligible, included in the study, completing follow-up, and analyzed – *N/A* |
|  |  | (b) Give reasons for non-participation at each stage – *N/A* |
|  |  | (c) Consider use of a flow diagram |
| Descriptive data | 14* | (a) Give characteristics of study participants (eg demographic, clinical, social) and information on exposures and potential confounders – *Page 4 - 5* |
|  |  | (b) Indicate number of participants with missing data for each variable of interest – *Page 6. Missing data only for reported cases.* |
| Outcome data | 15* | Report numbers of outcome events or summary measures – *Page 8* |
| Main results | 16 | (*a*) Give unadjusted estimates and, if applicable, confounder-adjusted estimates and their precision (eg, 95% confidence interval). Make clear which confounders were adjusted for and why they were included – *Page 9 - 10* |
|  |  | (*b*) Report category boundaries when continuous variables were categorized – *N/A* |
|  |  | (*c*) If relevant, consider translating estimates of relative risk into absolute risk for a meaningful time period – *N/A* |
| Other analyses | 17 | Report other analyses done—eg analyses of subgroups and interactions, and sensitivity analyses – *N/A* |
| Discussion | | |
| Key results | 18 | Summarise key results with reference to study objectives – *Page11* |
| Limitations | 19 | Discuss limitations of the study, taking into account sources of potential bias or imprecision. *Page 13* |
| Interpretation | 20 | Give a cautious overall interpretation of results considering objectives, limitations, multiplicity of analyses, results from similar studies, and other relevant evidence – *Pages 12-13* |
| Generalisability | 21 | Discuss the generalisability (external validity) of the study results – *Pages 13* |
| Other information | | |
| Funding | 22 | *N/A* |

*Give information separately for exposed and unexposed groups.
